# Supplementary material for: Skeletal descriptions of shape provide unique perceptual information for object recognition
Source: Sci Rep. 2019 Jun 27;9:9359. doi: 10.1038/s41598-019-45268-y (PMC6597715; doi:10.1038/s41598-019-45268-y)
Supplement: Supplementary file 1 — Supplemental Materials [file 41598_2019_45268_MOESM1_ESM.pdf]

Skeletal descriptions of shape provide unique perceptual information for object recognition

Vladislav Ayzenberg
Stella F. Lourenco

Supplemental Materials

Supplemental Experiment 1

One potential concern with our stimuli is that they may have been difficult to discriminate because they represent a single class of unfamiliar objects with a high degree of visual similarity. Such objects may be less likely to elicit the same mechanisms that support 'core' object recognition, which is thought to occur within 100-200 ms via a feedforward sweep through the ventral stream[1]. Instead, discrimination of these objects may elicit additional high-level processes, such as mental rotation, not typically implemented when discriminating familiar objects[2]. This possibility is difficult to rule out in the main experiments because participants were given unlimited time to discriminate between objects. Thus, in a supplemental experiment, we tested participants in a speeded task where the target object was presented for a 100 ms. If performance on this speeded task were comparable to performance on the unspeeded task (see Experiment 1 in the main text), then it would suggest that both tasks measure core object recognition.

Participants (n = 14) were administered a sequential match-to-sample task where they were asked to decide which of two choice objects matched a previously presented sample object. Each trial began with a fixation cross (500 ms), followed by a display with the sample object (100 ms), and then a display with two choice objects which remained onscreen until a response was made. One choice object had the same skeleton as the sample, and the other choice object had a different skeleton. The choice objects always had the same surface form as the sample (randomly selected) but were presented from different orientations (-30°, 0°, 30°). Participants were instructed to ignore the orientations of the objects and to make their decision on the basis of visual similarity. Each object was pitted against every other object an equal number of times (435 trials). Each object was approximately 6° × 6° in size, and choice objects subtended 9° from the center of the screen.

Comparisons to chance (0.50) revealed that participants were able to match the sample object with the correct choice object, M = 0.82% (M_{RT} = 946 ms), $t(13) = 26.8$, $p < .001$, $d = 7.16$, with 14/14 participants displaying accuracy above 0.74. This result suggests that our objects differed sufficiently to allow object recognition to occur within 100 ms.

In a subsequent analysis, we tested whether participants' performance on this task differed from their performance in the unspeeded discrimination task used in Experiment 1. We found that participants' performed comparably in the two tasks (M_{accuracy} = 0.82 vs. 0.80), with no statistical difference between groups (p = 0.46). These results are consistent with the speeded and unspeeded tasks recruiting similar perceptual processes, namely 'core' object recognition.

Supplemental Table 1. Linear regression results for each model used in Experiment 1.

Model	Standardized Coefficient		
	β	*t*	*p*
(Constant)		1.82	
Skeleton	0.27	6.01	< .001
Gabor-Jet	0.31	3.17	0.002
GIST	-0.18	-2.03	0.043
HMAX	0.11	2.06	0.040
AlexNet-fc6	0.13	1.80	0.073

Supplemental Table 2. Coefficients displaying the percentage of unique and shared variance explained by each model and model combinations, as well as percentages of the total explainable variance (20.5%) explained by each model and model combinations.

Model	Coefficient	Percentage of total
SKEL	6.631	33.305
GBJ	1.726	8.669
GIST	0.587	2.949
HMAX	0.732	3.678
fc6	0.499	2.508
SKEL + GBJ	-0.641	-3.218
SKEL + GIST	-0.241	-1.209
GBJ + GIST	-0.512	-2.569
SKEL + HMAX	-0.171	-0.859
GBJ + HMAX	0.344	1.730
GIST + HMAX	0.037	0.187
SKEL + fc6	1.005	5.046
GBJ + fc6	1.218	6.116
GIST + fc6	-0.105	-0.525
HMAX + fc6	0.445	2.235
SKEL + GBJ + GIST	0.224	1.125
SKEL + GBJ + HMAX	-0.095	-0.477
SKEL + GIST + HMAX	-0.009	-0.047
GBJ + GIST + HMAX	0.032	0.162
SKEL + GBJ + fc6	0.299	1.502
SKEL + GIST + fc6	-0.047	-0.237
GBJ + GIST + fc6	1.852	9.302
SKEL + HMAX + fc6	0.236	1.187
GBJ + HMAX + fc6	1.017	5.109
GIST + HMAX + fc6	-0.039	-0.196
SKEL + GBJ + GIST + HMAX	-0.006	-0.032
SKEL + GBJ + GIST + fc6	1.001	5.027
SKEL + GBJ + HMAX + fc6	0.037	0.186
SKEL + GIST + HMAX + fc6	0.009	0.045
GBJ + GIST + HMAX + fc6	3.084	15.493
SKEL + GBJ + GIST + HMAX + fc6	0.758	3.809

Supplemental Table 3. Random-effects regression results for each model used in Experiment 2. The standardized coefficients and *t*-values are drawn from a full regression model that included each model as a predictor. The χ^2 and *p*-values were calculated by iteratively testing the full regression model against ones without the predictor of interest.

Model	Standardized Coefficient			
	β	t	χ^2	p
(Intercept)		22.51		
Skeleton	-1.24	-4.80	22.30	< 0.001
Gabor-Jet	0.32	1.44	1.81	0.18
GIST	0.63	4.46	19.55	< 0.001
HMAX	-0.07	-0.50	0.19	0.66
AlexNet-fc6	0.06	0.21	0.05	0.83

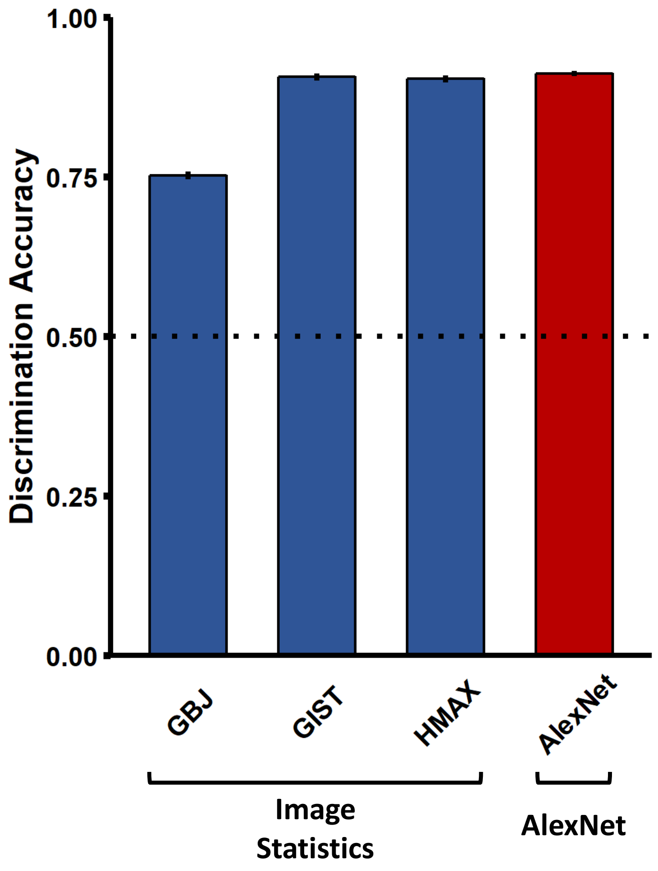

Supplemental Figure 1. Discrimination accuracies for all non-skeletal models. Each model was able to discriminate between objects significantly above chance (dotted line).

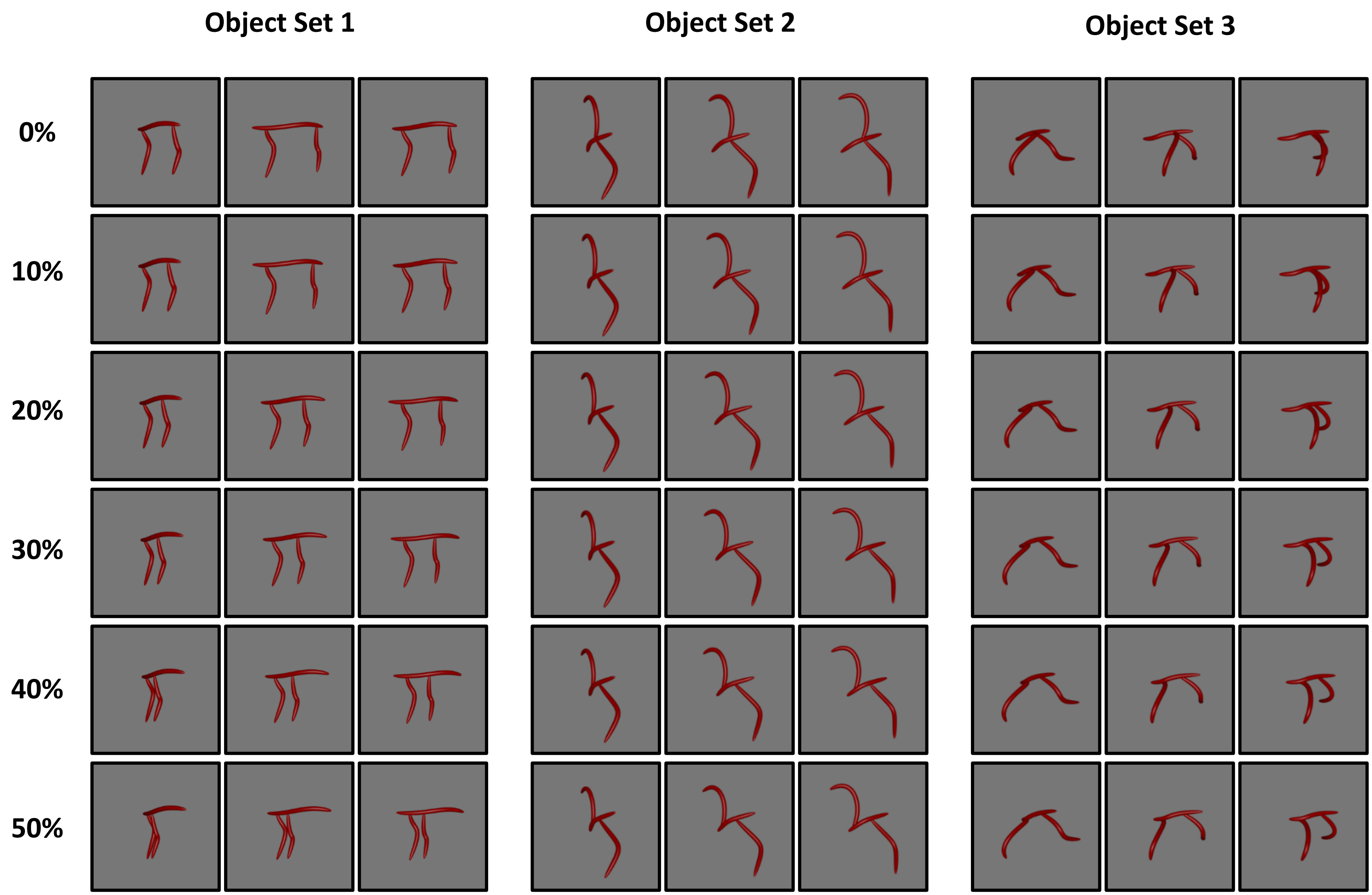

Supplemental Figure 2. All stimuli used in Experiment 2. Objects were comprised of three sets, each with distinct coarse spatial relations. Within each set, objects varied in skeletal similarity by increments of 0%, 10%, 20%, 30%, 40%, or 50% (each row). Each object could be presented in one of three orientations, each of which is depicted here (30°, 60°, 90°; each column within an object set).

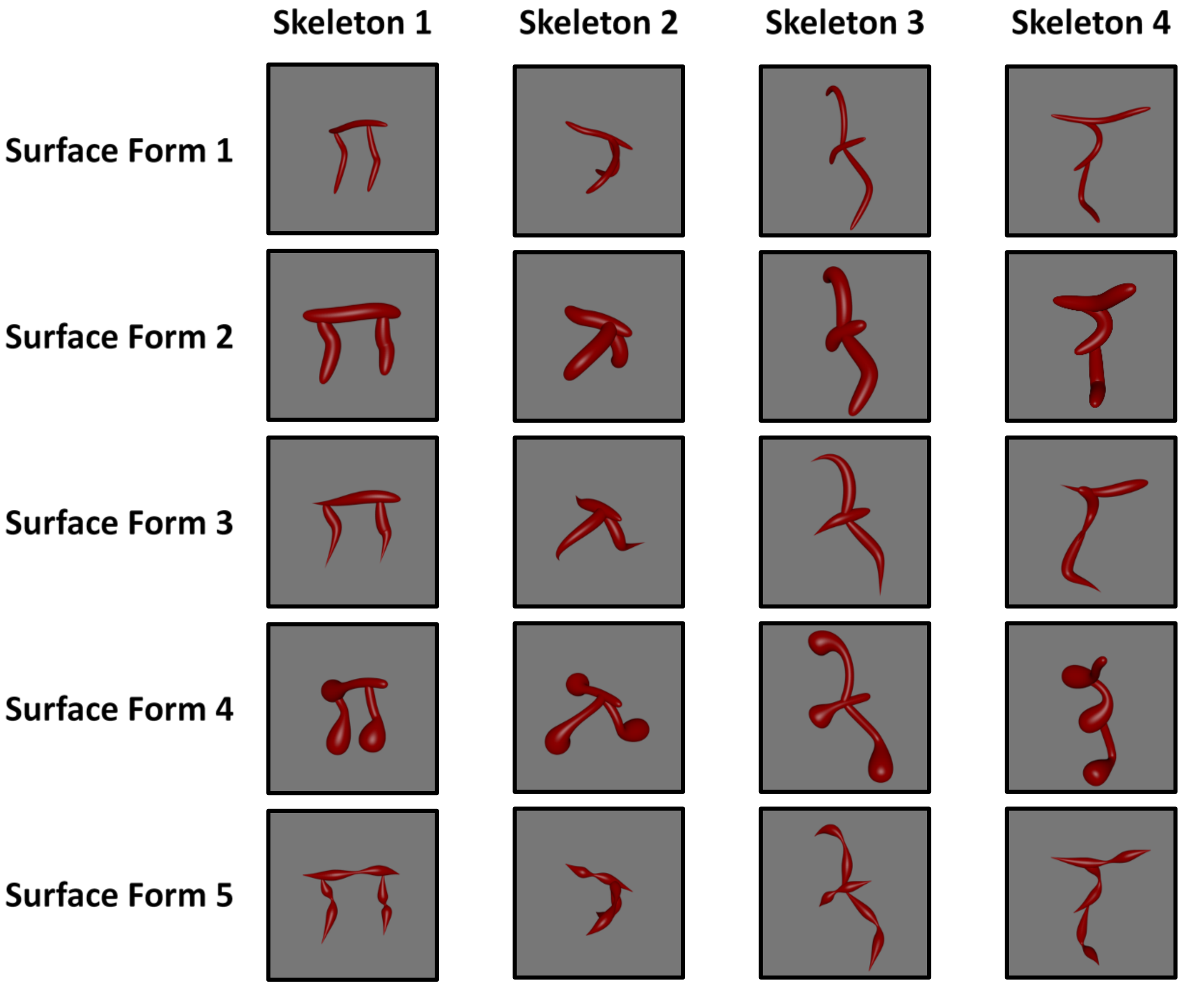

Supplemental Figure 3. The stimulus set used in Experiment 3. Each column displays objects with the same skeleton, but different surface forms. Each row displays objects with the same surface form, but different skeletons. Each object could be presented in one of three orientations (30°, 60°, 90°); a subset are depicted here.

References

1 Rajalingham, R. *et al.* Large-Scale, High-Resolution Comparison of the Core Visual Object Recognition Behavior of Humans, Monkeys, and State-of-the-Art Deep Artificial Neural Networks. *The Journal of Neuroscience* **38**, 7255-7269, doi:10.1523/jneurosci.0388-18.2018 (2018).

2 Gauthier, I. *et al.* BOLD activity during mental rotation and viewpoint-dependent object recognition. *Neuron* **34**, 161-171 (2002).
